# AtaA, a New Member of the Trimeric Autotransporter Adhesins from *Acinetobacter* sp. Tol 5 Mediating High Adhesiveness to Various Abiotic Surfaces

**DOI:** 10.1371/journal.pone.0048830

**Published:** 2012-11-14

**Authors:** Masahito Ishikawa, Hajime Nakatani, Katsutoshi Hori

**Affiliations:** Department of Biotechnology, Graduate School of Engineering, Nagoya University, Furo-cho, Chikusa-ku, Japan; University of Osnabrueck, Germany

## Abstract

*Acinetobacter* sp. Tol 5 exhibits an autoagglutinating nature and noteworthy adhesiveness to various abiotic surfaces from hydrophobic plastics to hydrophilic glass and stainless steel. Although previous studies have suggested that bacterionanofibers on Tol 5 cells are involved in the adhesive phenotype of Tol 5, the fiber that directly mediates Tol 5 adhesion has remained unknown. Here, we present a new member of trimeric autotransporter adhesins designated AtaA, which we discovered by analyzing a less adhesive mutant of Tol 5, T1, obtained by transposon mutagenesis. AtaA forms thinner and shorter nanofibers than fimbriae on Tol 5 cells. We performed target disruption of *ataA* by allelic marker exchange, and the resulting Δ*ataA* strain was complemented with *ataA* on the *Escherichia coli*-*Acinetobacter* shuttle vector, which was newly constructed. These results proved that AtaA is essential for Tol 5’s autoagglutinating nature and high adhesiveness to surfaces of various materials. In addition, the adhesiveness to solid surfaces mediated by AtaA is notably higher than that mediated by YadA of *Yersinia enterocolitica* WA-314. Moreover, and importantly, these characteristics can be conferred to the non-adhesive, non-agglutinating bacterium *Acinetobacter* sp. ADP1 *in trans* by transformation with *ataA*, with expected applications to microbial immobilization.

## Introduction

On bacterial cell surfaces, adhesive nanofibers, which are filamentous appendages of several nanometers in diameter, not only bind to host biomolecules and mediate infectious disease but also tether cells to abiotic surfaces [1]. Many specific interactions between pathogenic bacteria and host cells and tissues are mediated by proteinaceous “bacterionanofibers”, which can be divided into two major classes, fimbriae assembled from hundreds of subunits [2], [3], and non-fimbrial fibers having a simple monomeric or oligomeric structure [4].

Trimeric autotransporter adhesins (TAAs) belong to a subclass of non-fimbrial autotransporter adhesins and have recently attracted our attention as virulence factors of Gram-negative pathogens. TAAs form homotrimeric structures with a N-terminus-head-neck-stalk-membrane anchor-C-terminus architecture [5], [6]. The C-terminal anchor domain forms a 12-stranded β-barrel at the outer membrane (OM) and the head-neck-stalk domain, called a passenger domain, is secreted to the cell surface through the β-barrel pore. Although head and stalk domains alternate in the sequence of some TAAs, the anchor domain is always localized at the C-terminus, is homologous in all TAAs, and defines this family [7]. TAAs have been reported to mediate bacterial adhesion to host cells and/or extracellular matrix (ECM) proteins, such as collagen, fibronectin, and laminin, invasion of host cells, serum resistance, autoagglutination, and biofilm formation [4], [6], [8], [9], [10], [11], [12], [13], [14], [15], [16].

Generally speaking, nonspecific interactions between bacterionanofibers and abiotic surfaces are initially weak and reversible and enable many bacteria to crawl, glide, or twitch over solid surfaces [17]. Subsequently, bacterial cells attain strong and irreversible adhesion through interactions with surfaces at multiple sites of cell surface biomolecules, including nanofibers. Thereafter, during growth, bacteria on the surfaces form microcolonies, secrete exopolymeric substances (EPS), and develop biofilms, resulting in tenacious adhesion. Thus, bacterial cells require sufficient time to achieve tenacious adhesion to abiotic surfaces through formation of a biofilm. It has however been reported that in certain bacteria, such as *Caulobacter crescentus* with an adhesive extension of the cell envelope [18] and *Aggregatibacter actinomycetemcomitans* with Flp fimbria [19], nanofibers directly mediate rapid and tenacious adhesion to abiotic surfaces.

The toluene-degrading bacterium *Acinetobacter* sp. Tol 5 previously isolated from a biofiltration process demonstrated an autoagglutinating nature and noteworthy adhesiveness through the nanofibers on the cell surface [20], [21], [22]. Tol 5 cells are intrinsically adhesive to surfaces of various abiotic materials including hydrophobic plastics, hydrophilic glass, and stainless steel [23]. A large number of Tol 5 resting cells rapidly adhere to solid surfaces independently of cell growth. Tol 5’s initial attachment ability is quite high and distinguishable from its biofilm formation ability. At least three types of peritrichate nanofibers have been found on Tol 5 cells [24]. Interestingly, production of the peritrichate nanofibers was affected by the available growth substrate. Thick, long, straight nanofibers on cells grown on toluene, lactate, and ethanol were not observed on cells grown on triacylglycerol (TAG). In contrast, cells grown on TAG were covered with long, curved nanofibers, which only existed sparsely on cells grown on toluene, lactate, and ethanol. Thin, short, straight nanofibers were found densely covering the margin of cells grown on all four growth substrates. The analyses of cell adhesiveness and the expression levels of cell surface proteins relevant to the supplied carbon source suggested that the first and second types of nanofibers are type 1 and Fil fimbriae, respectively, and that these fibers are not responsible for the high adhesiveness of Tol 5.

To determine which nanofiber and which of its component molecules directly mediate adhesion of Tol 5 cells, we performed random gene disruption by transposon insertion [22]. Five strains that showed significantly decreased adhesiveness were obtained from culture suspension after cultivation of a mass of random insertion mutants in the presence of a polyurethane (PU) foam support. Of the five less-adhesive mutants obtained, T1 was the least adhesive. When Tol 5 cells were grown in the presence of the PU foam support, most of the wild type (WT) cells adhered to the support while T1 cells were individually dispersed in the culture broth. In the present study, we identified the gene disrupted in T1 as a new member of the TAA genes and demonstrated that this gene product is responsible for the high adhesiveness and autoagglutinating nature of Tol 5 cells.

## Results

### Discovery of a new TAA member in *Acinetobacter* sp. Tol 5

Transposon insertion sites on the chromosome of the less-adhesive mutant T1 were analyzed by Southern blot analysis using the Tn5 *tetA* gene [22] as a probe. On the chromosomal DNA of T1 digested with *Hin*dIII, a 5.1-kb fragment hybridized with the *tetA* probe. The fragment was sequenced, and the DNA sequence from the Tol 5 chromosome within the fragment was determined. The region of the Tol 5 WT chromosome adjacent to the transposon insertion site was amplified by inverse PCR and sequenced. Owing to the presence of long repeat sequences, it was difficult to determine the DNA sequence of the whole gene disrupted by Tn5 insertion. The sequencing strategy is described in Fig. S1. Finally, it was revealed that the structural gene encoding a protein belonging to the TAA family was disrupted in T1. This gene was designated *ataA* (*Acinetobacter* trimeric autotransporter adhesin), and its sequence data have been deposited with DDBJ/EMBL/GenBank under accession no. AB542908.

**Figure 1:**
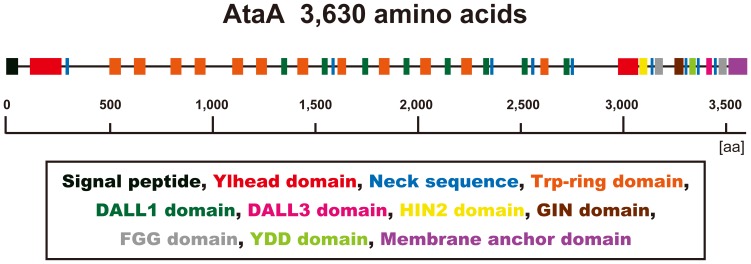
Predicted primary structure of AtaA annotated by the daTAA program ( http://toolkit.tuebingen.mpg.de/dataa **).** The colored regions represent annotated domains as indicated in the box.

AtaA is predicted to consist of 3,630 amino acid residues, which makes it one of the largest TAAs known to date (Fig. 1). Although AtaA follows the general N-terminus-head-stalk-anchor-C-terminus organization, an additional head domain localizes in the C-terminal region. The stalk domain of AtaA is notably longer than that of other TAAs and contains peptide repeats that are mosaically arranged. Although the boundaries of these repeat sequences are fuzzy, they can be divided into six groups when non-conserved amino acids are minimized in the respective groups; in each repeat sequence group, amino acid sequences are entirely conserved, or only a few amino acid residues are non-conserved (Fig. S2). Further domain annotation using the daTAA program [7] revealed that the two heads of AtaA were annotated as the Ylhead domain, the head of *Yersinia enterocolitica* YadA, which is the most well-studied TAA (Fig. 1). AtaA also has twelve Trp-rings, in which the tryptophan residue of the highly conserved Gly-Trp motif in many TAA Trp-ring domains is replaced by leucine or phenylalanine. As the Trp-ring consists mainly of β-meander motifs, the AtaA stalk domain is also anticipated to contain more β-structures than coiled coils, although coiled coils are abundant in most TAAs. In fact, in the ultrastructure of AtaA, coiled coils were predicted to be infrequent at the N-terminal stalk domain according to analysis performed using the COILS program (Fig. S3) [25].

**Figure 2:**
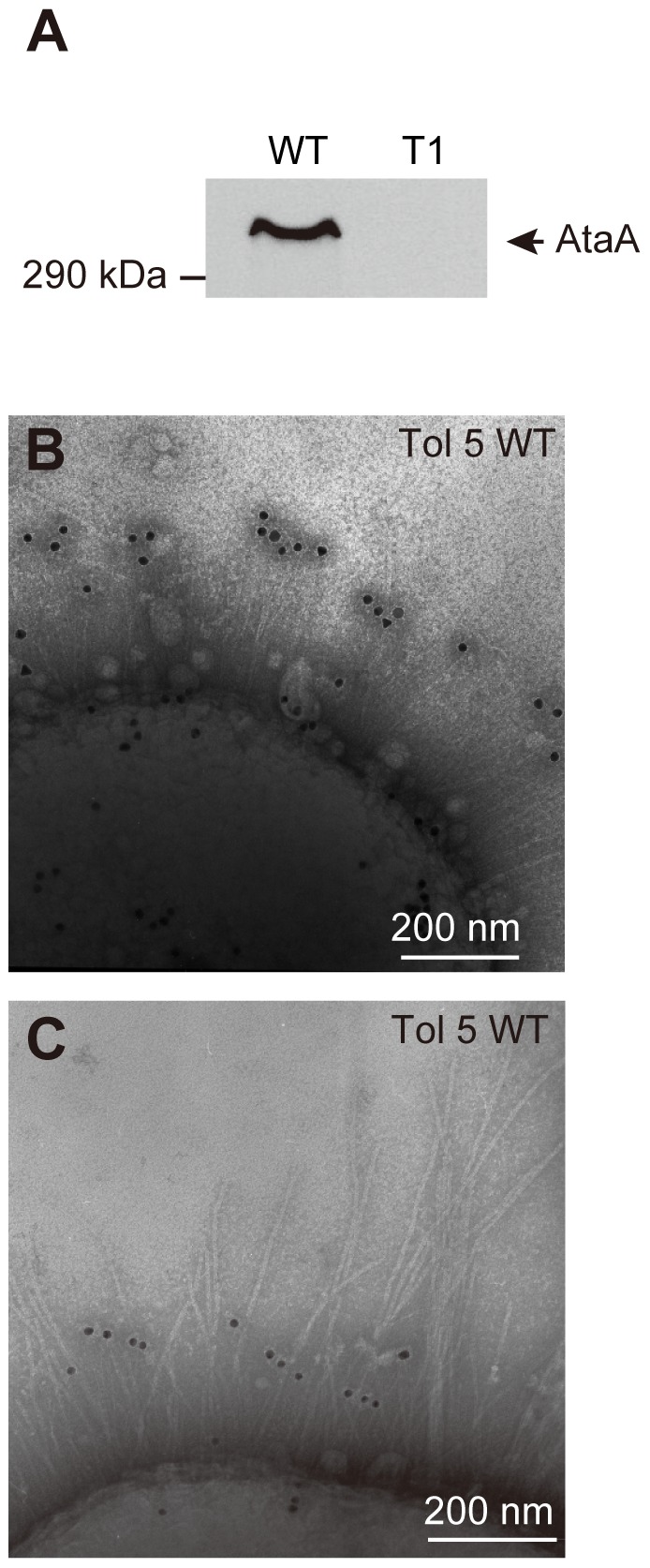
Detection of AtaA protein and fiber by Western-blot analysis and immunoelectron microscopy. (**A**) Immunodetection of AtaA using anti-AtaA antiserum against outer membrane (OM) proteins prepared from Tol 5 WT and Tol 5 T1 (T1). (**B–C**) *Acinetobacter* sp. Tol 5 observed by immunoelectron microscopy using an anti-AtaA antibody. (**B**) Thin peritrichate nanofibers on Tol 5 WT cells were labeled with anti-AtaA_699–1014_ antibody. (**C**) The anti-AtaA_699–1014_ antibody specifically binds to the thinnest, shortest nanofibers on Tol 5 cells.

To identify which nanofiber is composed of AtaA, Western-blot analysis and immunoelectron microscopy were carried out using an anti-AtaA antibody against a part of the stalk domain (AtaA_699–1014_). AtaA expression and depletion were confirmed at the OM of the Tol 5 WT and the mutant strain T1, respectively (Fig. 2A). Immunoelectron microscopy revealed that AtaA composes the thinnest and shortest nanofiber (200 nm long, 3.5±0.4 nm diameter) of the three types of peritrichate nanofibers previously reported [24] (Fig. 2B and 2C). The antibody labels did not line each fiber but were localized solely near the distal end of the fiber, showing that AtaA architecture is different from fimbriae constructed by the accumulation of a single protein in the hundreds. In our previous study, the larger nanofibers, which often obscured the smallest AtaA fibers, were deduced to be dominant type 1 fimbriae and sparse Fil fimbriae [24]. Furthermore, the lack of AtaA fibers on T1 was confirmed by immunoelectron microscopy (Fig. S4).

### Involvement of AtaA in the high adhesiveness of Tol 5 cells

**Figure 3:**
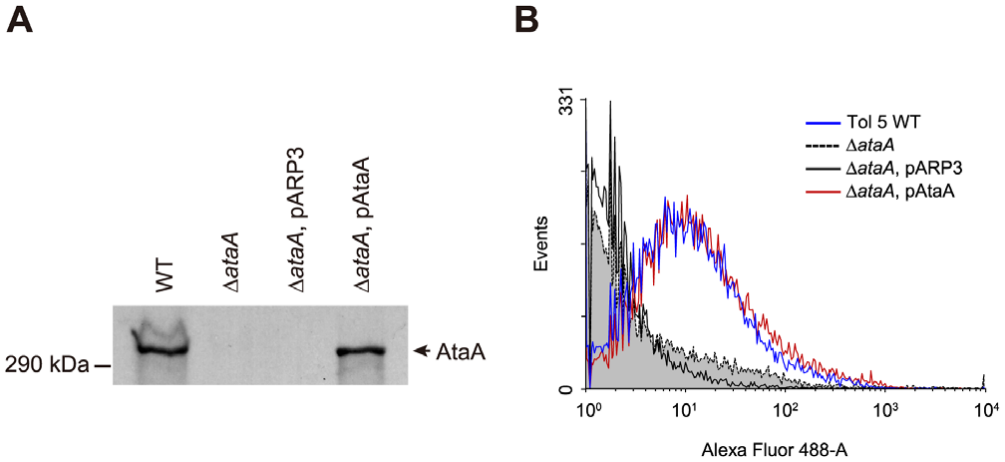
Detection of AtaA protein at the OM and flow cytometry analysis for the quantification of displayed AtaA on the cell surface. (**A**) Immunodetection of AtaA using anti-AtaA antiserum against OM proteins prepared from Tol 5 WT, Δ*ataA*, Δ*ataA* transformed with the empty vector pARP3 (Δ*ataA*, pARP3), and Δ*ataA* complemented with *ataA* in pARP3 (Δ*ataA*, pAtaA). (**B**) Confirmation of surface-displayed AtaA by flow cytometry. Δ*ataA*, pAtaA (red line) shows AtaA proteins in a level similar to Tol 5 WT (blue line) on its cell surface. Δ*ataA* (dashed line, gray fill) and Δ*ataA*, pARP3 (black line) strains are shown as negative controls.

To clarify that disruption of *ataA* causes a lack of high adhesiveness in Tol 5 cells, allelic marker exchange was carried out for target disruption of *ataA*. The resulting Δ*ataA* mutant was confirmed to neither produce AtaA protein at the OM by Western-blot analysis (Fig. 3A) nor display AtaA fibers on the cell surface by immunoelectron microscopy (Fig. S4) and flow cytometry (Fig. 3B). Expression levels of components of type 1 fimbriae and Fil fimbriae, as well as respective fiber production in the Δ*ataA* mutant, were also confirmed to be similar to those in the WT (Fig. S5).

**Figure 4:**
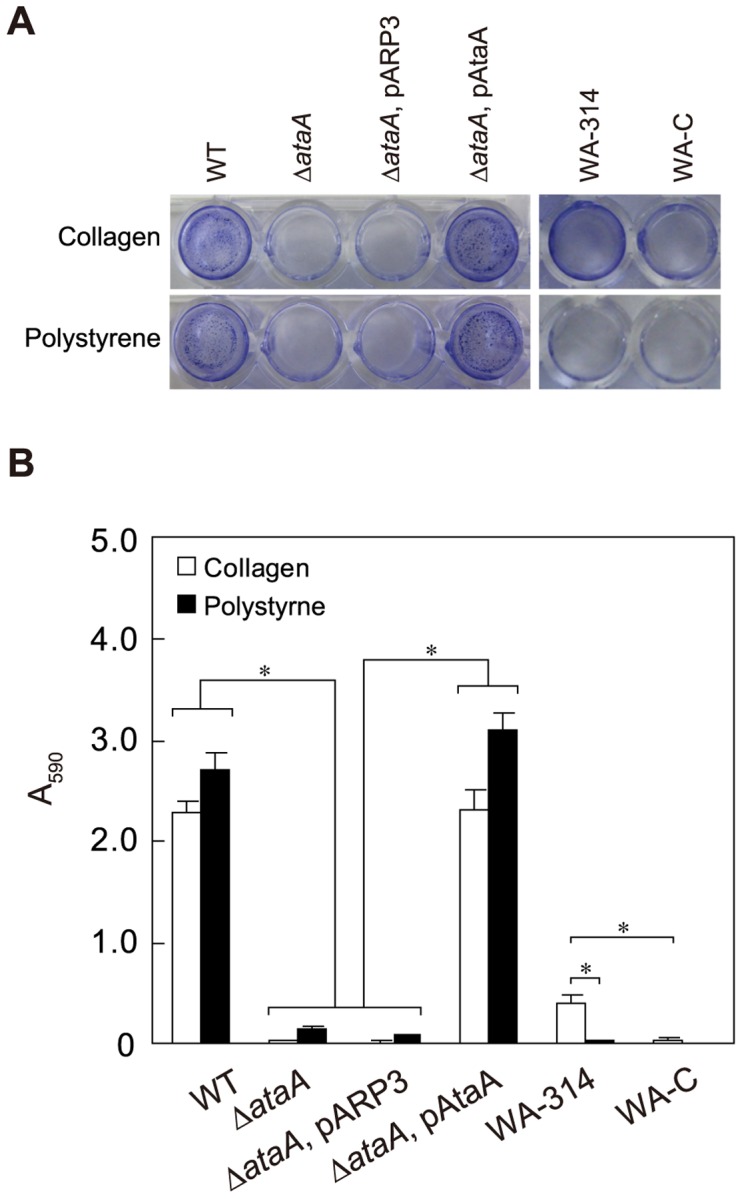
Adherence assay of *Acinetobacter* sp. Tol 5, its derivatives, and *Y. enterocolitica* strains. (**A**) Bacterial cells adhering to 48-well polystyrene (PS) plates or type I collagen-coated plates were stained with crystal violet. Strains expressing *ataA* (WT; Δ*ataA*, pAtaA) adhere to both type I collagen-coated and PS surfaces, while Tol 5 strains lacking functional *ataA* (Δ*ataA*; Δ*ataA*, pARP3) cannot adhere to either surface. *Y. enterocolitica* expressing YadA (WA-314) specifically adheres to the collagen surface, while YadA-deficient derivatives of WA-314 (WA-C) barely adhere to both surfaces. (**B**) The adherence of cells stained with crystal violet in (A) was quantified by measuring the absorbance at 590 nm (A_590_) of an ethanol solution used to dissolve the stain. White and black bars denote adherence to the collagen surface and the PS surface, respectively. Data are expressed as mean and SEM (n = 3) values. Statistical significance, ^*^P<0.01.

Figure 4 shows the results of the adherence assay using 48-well polystyrene (PS) plates and type I collagen-coated PS plates under a resting cell condition. Although the photographs resemble those of a biofilm formation assay, the adherence assay in the present study differed from a biofilm formation assay and directly reflected the initial attachment ability of bacteria to solid surfaces, as reported previously [23]. To compare adhesiveness mediated by AtaA with that mediated by a typical TAA, two *Y. enterocolitica* strains, WA-314 and WA-C, were subjected to the adherence assay with Tol 5 and its derivative strains. The strain WA-314 expresses YadA and exhibits YadA-dependent adhesion to type I collagen [26], whereas the strain WA-C is a YadA-deficient derivative of WA-314 formed by curing of the virulence plasmid pYVO8 [27]. Tol 5 WT cells showed high adhesiveness to both surfaces, regardless of collagen-coating. Although the strain WA-C scarcely adhered to both surfaces, the strain WA-314 expressing YadA preferred the collagen-coated surface (P<0.01) to the abiotic surface; but its adhesiveness was trivial compared with Tol 5 WT cells. Cells of the Δ*ataA* mutant adhered only slightly to both surfaces, implying that the deficiency of AtaA nanofibers results in the poor adhesiveness of Tol 5 cells.

For further confirmation of AtaA involvement in Tol 5 adhesion, we tried to complement the Δ*ataA* mutant with *ataA* on a plasmid vector. However, Tol 5 cells were difficult to transform due to their low competency, we constructed the novel *Acinetobacter-Escherichia coli* shuttle vector pARP3. This expression plasmid vector can be introduced into recipients by conjugation, is capable of replicating in *Acinetobacter* strains, and possesses an arabinose-inducible promoter (Fig. S6). The expression level of a gene may be important when the physiology of a bacterium (e.g., pathogenicity) is the primary focus, because the responsibility of the target gene for physiology under natural conditions is important. However, when functions or properties of gene products themselves are the focus, researchers demonstrate the functions and properties under elicitable conditions. In fact, for the characterization of individual TAAs, other researchers on TAAs also have used inducible promoters [13], [28], [29], [30]. Thus, the *ataA* locus containing the Shine-Dalgarno (SD) sequence was cloned under the control of the arabinose-inducible promoter of pARP3, generating the *ataA*-expression vector pAtaA. The Δ*ataA* mutant was transformed with pARP3 (Δ*ataA*, pARP3) or pAtaA (Δ*ataA*, pAtaA), and the OMs of these transformants were subjected to Western-blot analysis. Although no AtaA was detected at the OM of Δ*ataA*, pARP3 cells (negative control), Δ*ataA*, pAtaA cells restored AtaA production to a similar level as that in the WT (Fig. 3A). These transformants were also subjected to flow cytometry to analyze the surface display of AtaA. The resultant histograms of flow cytometry showed that AtaA displayed on the cell surface of the complemented mutant (Δ*ataA*, pAtaA) was restored to a level similar to that in the WT (Tol 5 WT), regardless of the use of the inducible promoter in the multiple copied plasmid (Fig. 3B). The adherence assay of the complemented mutant, whose *ataA* expression level was almost equal to that of the WT, demonstrated that complementation of Δ*ataA* with pAtaA entirely restored the high adhesiveness to both PS and collagen surfaces (Fig. 4). These results prove that AtaA is essential for the high adhesiveness of Tol 5, that the AtaA-mediating adhesiveness is notably higher than the YadA-mediating adhesiveness, and that AtaA interacts with abiotic surfaces to at least the same degree as it does with a biotic collagen surface.

### Adhesive features mediated by AtaA

**Figure 5:**
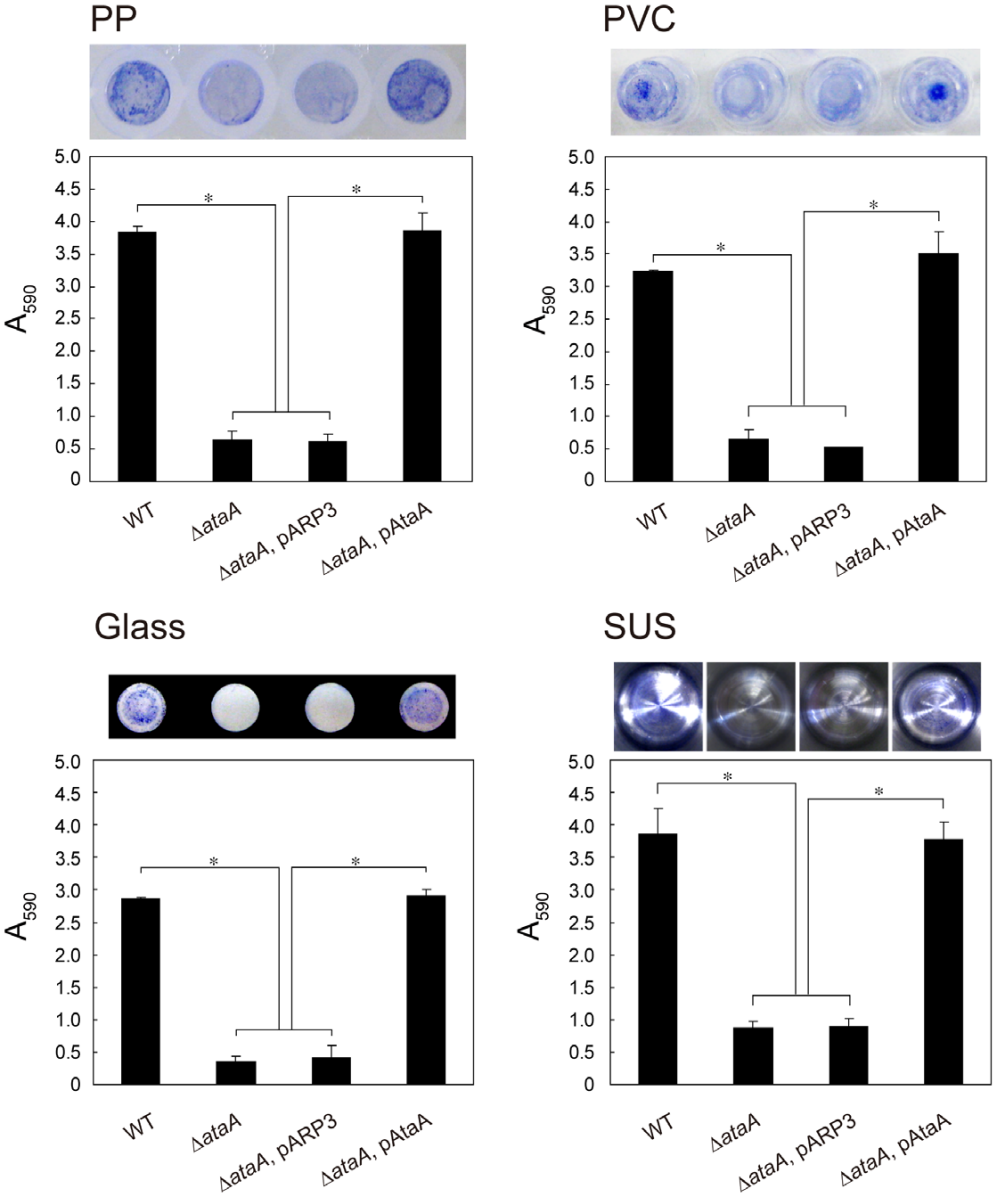
Adhesion of *Acinetobacter* sp. Tol 5 and its derivatives to different abiotic surfaces. Adhesion of Tol 5 and its derivatives (Δ*ataA*; Δ*ataA*, pARP3; Δ*ataA*, pAtaA) to 96-well plates made of polypropylene (PP), polyvinylchloride (PVC), and glass, and to a sample cup made from stainless steel (SUS) was assessed by the same procedure as the adherence assay for a 48-well PS plate. Photographs indicate the stained cells adhering to well surfaces. Data are expressed as mean and SEM (n = 3) values. Statistical significance, ^*^P<0.01.

The Tol 5 strain, despite its hydrophobic cell surface, can adhere to hydrophilic glass and metal surfaces as well as to hydrophobic plastic surfaces [23]. To confirm that this nonspecific adhesiveness is mediated by AtaA, we examined the adhesiveness of the Δ*ataA* mutant, its transformant with the empty vector pARP3 (Δ*ataA*, pARP3), and the complemented mutant (Δ*ataA*, pAtaA) to four abiotic surfaces: polyvinylchloride (PVC), polypropylene (PP), glass, and stainless steel (SUS). While Δ*ataA* and Δ*ataA*, pARP3 strains showed low adhesiveness to these surfaces, the Δ*ataA*, pAtaA strain restored the nonspecific and high adhesiveness of Tol 5 WT, indicating that AtaA is responsible for the nonspecific adhesion of Tol 5 cells (Fig. 5).

**Figure 6:**
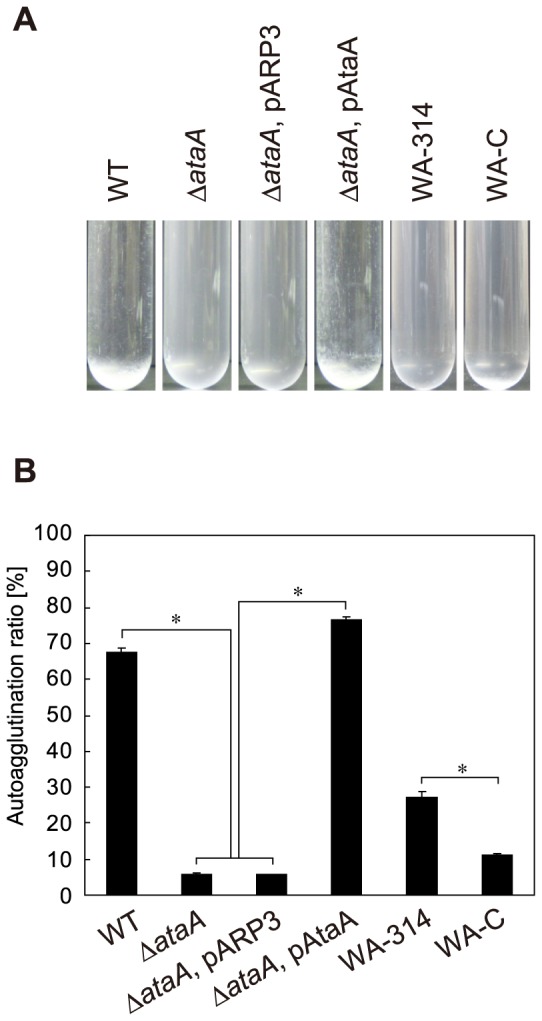
Autoagglutination assay of *Acinetobacter* sp. Tol 5, its derivatives, and *Y. enterocolitica* strains. (**A**) Strains expressing *ataA* (WT; Δ*ataA*, pAtaA) autoagglutinate and form cell clumps that settle at the bottom of test tubes. After standing for 3 h without agitation, suspensions of these strains were transparent due to the sedimentation of cells, whereas cell suspensions of strains lacking functional *ataA* (Δ*ata*A; Δ*ataA*, pARP3) remained cloudy. *Yersinia* strain expressing YadA (WA-314) partially formed cell clumps. Cell suspension of WA-314 strain was slightly transparent compared with the *yadA*-mutant strain (WA-C). (**B**) Autoagglutination observed in (A) was quantified by a decrease in optical density at 660 nm (OD_660_). Graph bars show the autoagglutination ratio (%). Data are expressed as mean and SEM (n = 3) values. Statistical significance, ^*^P<0.001.

T1 cells obtained by Tn5 random insertion not only show low adhesiveness but also lack the autoagglutinating nature of Tol 5 [31], and some TAAs are involved in autoagglutination, which is self-adhesion of cells [6]. Therefore, this feature was also examined for the Δ*ataA* mutant and its derivatives. The cell suspension was prepared by the same procedure as the adherence assay to prevent cell growth and subjected to the tube-settling assay to quantify the sedimentation of cell clumps. While the WT and Δ*ataA*, pAtaA strains showed high autoagglutination ratios, these ratios of Δ*ataA* and Δ*ataA*, pARP3 remained very low during the course of the assay (Fig. 6). Although strain WA-314 showed a higher autoagglutination ratio than strain WA-C, the value was much lower than that of Tol 5. Therefore, AtaA mediates the autoagglutination as well as the high adhesiveness of *Acinetobacter* sp. Tol 5.

### Expression of *ataA* in another non-adhesive, non-agglutinating bacterium

**Figure 7:**
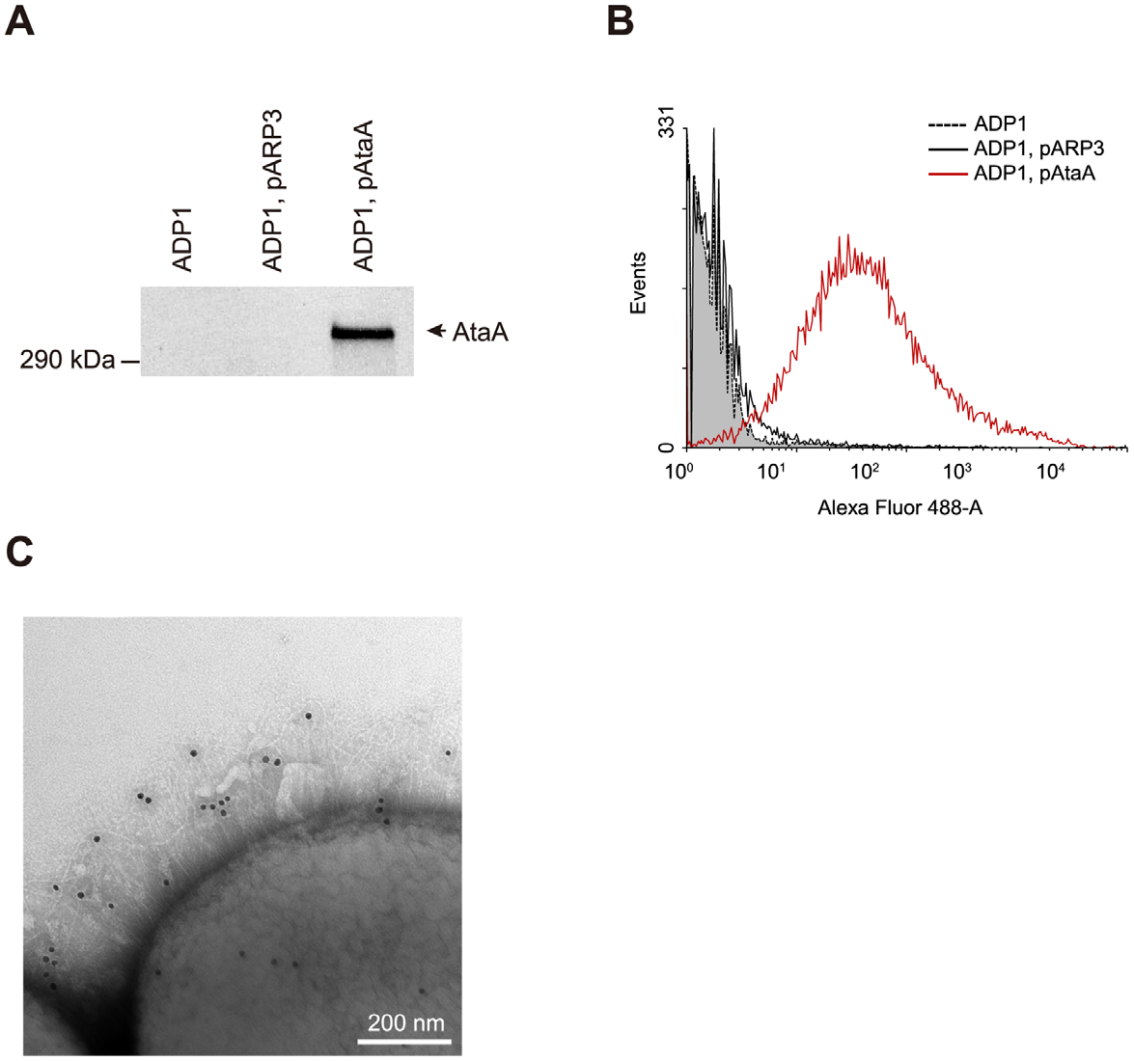
Expression of *ataA* in another *Acinetobacter* strain, ADP1. (**A**) Immunodetection of AtaA using anti-AtaA antiserum against OM proteins prepared from *Acinetobacter* sp. ADP1, ADP1 transformed with the empty vector pARP3 (ADP1, pARP3), and ADP1 transformed with *ataA* in pARP3 (ADP1, pAtaA). (**B**) Flow cytometry analysis of AtaA displayed on the cell surface of ADP1 and its transformants. ADP1, pAtaA (red line) shows a large number of AtaA proteins on its cell surface. ADP1 (dashed line, gray fill) and ADP1, pARP3 (black line) strains are shown as negative controls. (**C**) Confirmation of AtaA fibers on strains expressing *ataA* (ADP1, pAtaA) by immunoelectron microscopy.

To conclude that the adhesive features described above are intrinsic in nature of AtaA, we finally examined the capability of AtaA to confer the adhesive features to another strain of Gram-negative bacteria. Another non-adhesive, non-agglutinating *Acinetobacter* species, strain ADP1, which is useful for biotechnological applications due to its capability to degrade and produce many important chemicals [32], was transformed with pAtaA. Since this strain is highly competent for natural transformation, resulting in ease of genetic modification of this strain [33], it is an ideal model organism for genome engineering and is superior to *Escherichia coli* in this respect [34]. The resultant transformant (ADP1, pAtaA) produced AtaA protein at the OM (Fig. 7A) and displayed AtaA nanofibers on ADP1 cells (Fig. 7B, C). Thus, we succeeded in the production of AtaA nanofibers on cells of a different bacterial strain from the original Tol 5.

**Figure 8:**
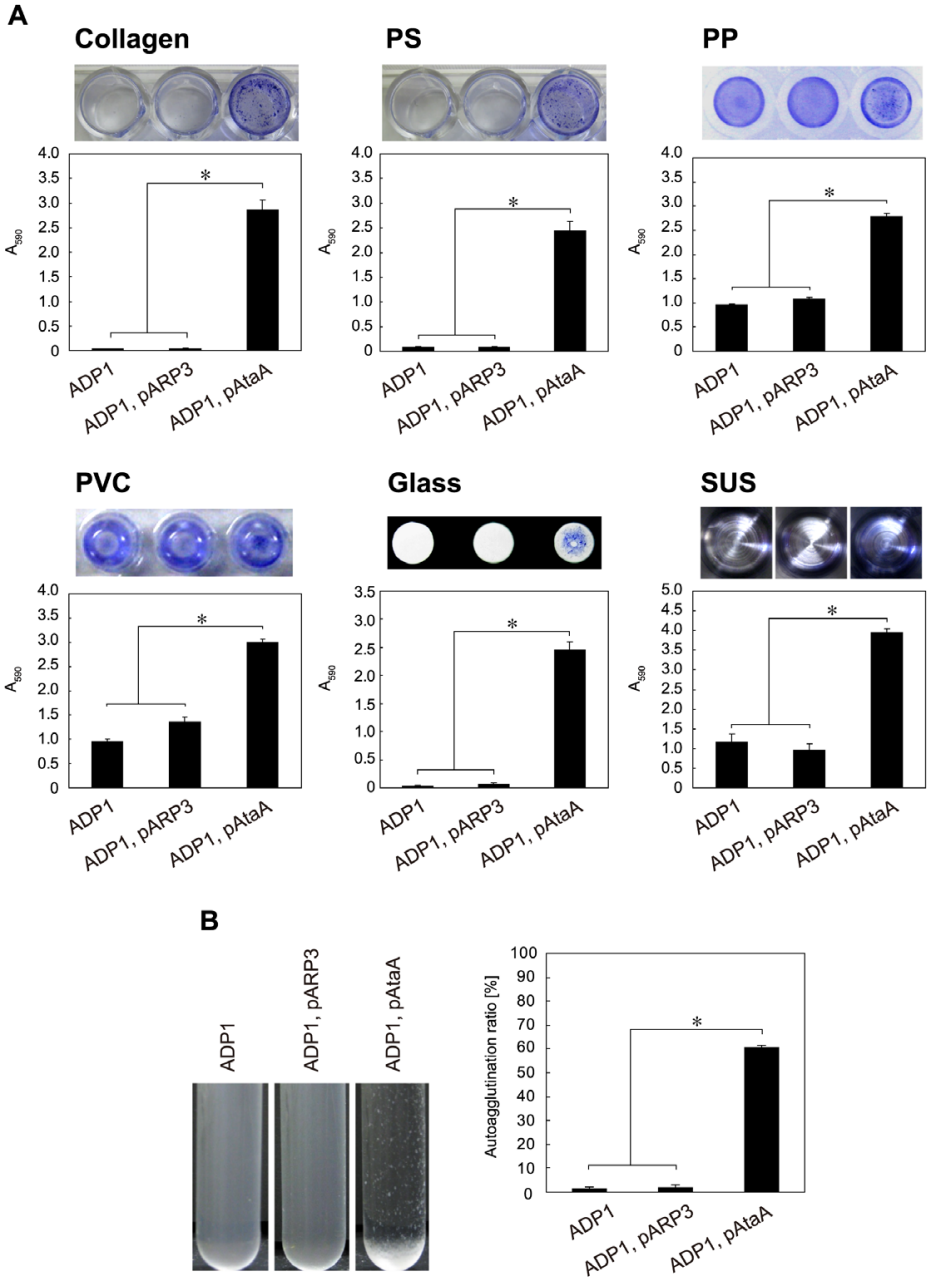
Adherence and autoagglutination assays of *Acinetobacter* sp. ADP1 and its transformants. (**A**) Adherence assay of ADP1 and its transformants (ADP1; ADP1, pARP3; ADP1, pAtaA) to 48-well PS and type I collagen-coated plates, to 96-well plates made of PP, PVC, and glass, and to a sample cup made from SUS. Data are expressed as mean ± SEM (n = 3) values. Statistical significance, ^*^P<0.01. (**B**) Autoagglutination assay of ADP1 and its transformants (ADP1; ADP1, pARP3; ADP1, pAtaA). Graph bars show the autoagglutination ratio (%). Data are expressed as mean ± SEM (n = 3) values. Statistical significance, ^*^P<0.001.

The ADP1, pAtaA strain, the original ADP1, and the transformant harboring the empty vector pARP3 (ADP1, pARP3) were evaluated for their adhesiveness to various solid surfaces composed of biotic collagen, hydrophobic plastics, hydrophilic glass, and hydrophilic metal, as described above for Tol 5 and its derivatives. Although we previously reported the adhesion preferences of ADP1 for PP, PVC, and SUS surfaces [23], strain ADP1, pAtaA showed nonspecific adhesion to all examined surfaces (Fig. 8A). Exogenous AtaA conferred the high adhesiveness of ADP1 cells to type I collagen, PS, and glass surfaces in a level similar to that of Tol 5 cells, although the original ADP1 cells could adhere only slightly or not at all to these surfaces. The adhesiveness of ADP1 cells to PP, PVC, and SUS surfaces was also elevated by AtaA to a level comparable with that of Tol 5 in relation to the adhesiveness of the original cells, which was less than half or one-third that of Tol 5.

ADP1 and its derivatives were also subjected to the tube-settling assay for quantification of autoagglutination. ADP1 cells intrinsically showed a non-agglutinating nature, but the ADP1, pAtaA strain acquired the autoagglutinating nature through the expression of exogenous *ataA* (Fig. 8B). Thus, it was proven that AtaA has a nature of exhibiting high adhesiveness to various solid surfaces and autoagglutinating bacterial cells and that this nature could be conferred *in trans* to another bacterial strain.

## Discussion

In the present study, we discovered AtaA, a new member of the TAA family, in *Acinetobacter* sp. Tol 5, which intrinsically exhibits a high adhesive phenotype to various abiotic surfaces. Most reports on TAAs have focused on their ability to adhere to biotic surfaces, although several TAAs have been reported to be responsible for biofilm formation on various abiotic surfaces [8], [13], [29], [35], [36]. However, biofilm formation ability is different from the initial attachment ability exhibited by AtaA [23]. Biofilm formation includes multiple biological factors as well as the direct interaction between adhesins and surfaces. During formation of biofilms, EPS, which contains a variety of biopolymers, such as polysaccharides, proteins, and DNA, are secreted from bacterial cells and construct the matrix of biofilms. Furthermore, involvement of TAAs in biofilm formation on abiotic surfaces might be attributed to the autoagglutination function rather than to initial attachment, as reported in UpaG of *E. coli* [13]. Recently, Müller et al analyzed the initial attachment of bacterial cells to solid surfaces via three TAAs, BadA of *Bartonella henselae*, Vomps of *Bartonella quintana*, and YadA of *Y. enterocolitica*, under static and flow conditions [37]. In their study, the TAAs, particularly BadA, mediated relatively nonspecific adherence to “biotic” surfaces as well as adherence to a plastic surface under the static condition, although YadA did not mediate adherence to fibronectin surfaces. Our data demonstrate that adhesiveness mediated by AtaA to “abiotic” surfaces is nonspecific and notably higher than that mediated by YadA to both PS and type I collagen surfaces. Furthermore, and importantly, this phenotype can be conferred to other bacterial species by introducing the single gene *ataA*. This potential leads us to expect that AtaA can be utilized for direct immobilization of bacterial cells to abiotic support surfaces.

The most frequently used technique for microbial immobilization involves entrapping cells in polymer gel; however, such an approach adversely affects the mass transfer rate in the inner part of the gel [38], [39]. Therefore, direct immobilization of bacterial cells onto the surface of a support by utilizing AtaA would represent a great advance for bioengineering employing whole microbial cells. In theory, the introduction of AtaA into industrially relevant Gram-negative bacteria, including other *Acinetobacter* strains, would provide a novel and effective means for the immobilization of bacteria in the production of chemicals, biomass energy, and other applications.

Our data demonstrate that AtaA also mediates autoagglutination (Figs. 6 and 8B). Autoagglutination has been reported to be mediated by various types of nanofibers and to be important for biofilm formation on both biotic and abiotic surfaces [3], [6], [13], [15], [40]. However, it is regarded as a form of specific binding since it is mediated by homologous recognition of an adhesin or interaction with other target molecules on the cell surface [6], [15]. The functional sites mediating the high adhesiveness of AtaA to solid surfaces and those for autoagglutination may be different.

With regard to features of the primary structure of AtaA, multiple head domains are seen not only in AtaA but also in some TAAs [13], [41], [42], [43], [44]. Although mosaically arranged repeat sequences are also a common feature of TAAs [7], the AtaA stalk that consists of extremely conserved, long (58–243 residues) repeats is remarkable. Recently, Cha adhesin in *Haemophilus* cryptic genospecies was also found to contain a series of entirely conserved tandem repeats, but they consist of only 28 residues in contrast with the long repeat sequences in AtaA [12]. Although the tandem repeats in Cha affect the adherence activity of the *Haemophilus* strain [44], the function of the long repeat sequences in AtaA remains to be studied.

On the basis of our results alone, the possibility that other proteins cooperate with AtaA in Tol 5 adhesion cannot be denied. However, other bacterial species that are intrinsically non-adhesive and non-autoagglutinating acquired the adhesive properties by introduction of the single gene *ataA*. This strongly suggests that AtaA is the primary mediator of the high adhesiveness and autoagglutinating nature of bacteria.

## Materials and Methods

### Culture conditions

The bacterial strains used in this study are listed in Table 1. *Acinetobacter* sp. Tol 5 and its derivative mutants were grown in basal salt (BS) medium at 28°C as described previously [22] or in Luria-Bertani (LB) medium. *Acinetobacter* sp. ADP1 and its derivative mutants were grown in LB medium at 30°C. Antibiotics were used at the following concentrations when required: ampicillin (1,000 µg/ml), tetracycline (10 µg/ml), gentamicin (10 µg/ml) for Tol 5 derivative mutants; ampicillin (100 µg/ml) and gentamicin (10 µg/ml) for ADP1 derivative mutants. Arabinose was added to a final concentration of 0.5% (wt/vol) for the induction of *ataA* under the control of the P*_BAD_* promoter. For expression of YadA, *Y. enterocolitica* was grown overnight in LB medium at 27°C, the overnight culture was diluted to 1∶20 in LB medium, and then incubated at 37°C for 3 h, as described previously [26].

**Table 1:** Bacterial strains and plasmids used in this study.

Strain or plasmid	Description^a^	Reference
*Acinetobacter* sp.		
Tol 5	Wild type strain	[20]
Tol 5 T1	Tol 5 mutant lacking adhesive and autoagglutinating properties	[22]
*ΔataA*	Tol 5 mutant with defect in *ataA* by allelic marker exchange	This study
ADP1	A representative strain of *Acinetobacter*	[33]
*E. coli*		
DH5α	Host for routine cloning	Takara
DH10B	Host for routine cloning	Invitorogen
S17-1	Donor strain for conjugation	[45]
BL21	Host for protein expression	GE Healthcare
*Y. enterocolitica*		
WA-314	*Y. enterocolitica* serotype O:8 expressing YadA	[27]
WA-C	Plasmidless derivative of WA-314, YadA^−^	[27]
Plasmid		
pUC118	Cloning vector, Ap^r^	Takara
pCR-XL-TOPO	Cloning vector, Km^r^	Invtirogen
pDONR221	Donor vector for Gateway technology, Km^r^	Invtirogen
pGEX4T-2	Expression vector for GST fusion protein, Ap^r^	GE healthcare
pWH1266	*E. coli-Acinetobacter* shuttle plasmid, Tc^r^, Ap^r^	[47]
pJQ200sk	Mobile plasmid, SacB, Gm^r^	[46]
pP3	Source of P3-Ap^r^ cassette; high resistance to ampicilin, Km^r^, Ap^r^	T. Kurihara
pSAE1	pJQ200sk::*Acinetobacter* origin, SacB, Gm^r^	This study
pSAE2	pSAE::*Pvu*II site, ΔSacB, Gm^r^	This study
pSAE3	pSAE::P3-Ap^r^, ΔSacB, Gm^r^, Ap^r^	This study
pJN105	Source of *araC*-P*_BAD_* system, Gm^r^	[48]
pARP3	*E. coli*-*Acinetobacter* shuttle expression vector, pSAEΔSacB::P3 Ap^r^::*araC*-P*_BAD_*, Gm^r^, Ap^r^	This study
pAtaA	*ataA*-expression vector, pARP3::*ataA*	This study

a Ap, ampicillin; Km, kanamycin; Tc, tetracycline; Gm, gentamycin.

### DNA manipulation

General DNA manipulations, such as PCR, restriction enzyme digestion, and ligation, were performed using standard protocols. The plasmids and the primers used in this study, with the exception of those used for primer walking during DNA sequencing, are detailed in Table 1 and Table S1, respectively. Transformation of Tol 5 T1 was carried out by bacterial conjugation with donor strain *E. coli* S17-1 harboring the target plasmid [45]. Transformation of *Acinetobacter* sp. ADP1 was performed by natural transformation as described previously [34].

### Construction of pARP3 and pAtaA

A mobile plasmid, pJQ200sk [46], was used as the plasmid backbone. A fragment containing an *Acinetobacter* origin of replication was amplified by PCR from pWH1266 [47] using the primers *Acineto*-oriF/*Acineto*-oriR, digested with *Sph*I, and then ligated into the *Sph*I site of pJQ200sk to generate pSAE1. To remove the *sacB* gene and introduce a new *Pvu*II site in pSAE1, inverse PCR was performed using the primers dele-sacBF/dele-sacBR, and the amplified PCR product was digested with *Pvu*II and then self-ligated to generate pSAE2. Although Tol 5 and T1 have potential ampicillin resistance, both are sensitive to high concentrations of ampicillin (the minimum inhibitory concentration for growth is 120 µg/ml). The Tol 5 transformant harboring plasmid pP3, which expresses the *bla* gene under the control of promoter P3 and confers resistance to high concentrations of ampicillin, could survive in LB medium containing 1,000 µg/ml ampicillin. The P3-Ap^r^ cassette was amplified from pP3 by PCR using the primers p3-ApF/p3-ApR, digested with *Pvu*II, and then cloned into the *Pvu*II site of pSAE2 to generate pSAE3 which facilitated selection of Tol 5 derivative transformants. The *araC*-P*_BAD_* system was excised from the pJN105 plasmid [48] as a *Sac*I-*Kpn*I fragment and cloned into the *Sac*I-*Kpn*I site of the pSAE3, generating pARP3 (Fig. S6), which served as an arabinose-inducible expression vector capable of replicating in *Acinetobacter* strains.

Cloning *ataA* under the control of promoter P*_BAD_* of pARP3 was performed in two steps. First, the fragment containing the deduced Shine-Dalgarno (SD) sequence and the entire *ataA* gene was amplified by PCR using two primer sets, attB1-*ataA*F/attB2-*ataA*R and attB1-adapter/attB2-adapter. The PCR product was inserted into pDONR221 using BP Clonase II enzyme mix (Invitrogen), and both the SD sequence and the entire *ataA* gene were then inserted into the *EcoR*I-*Xba*I site of pARP3 as an *Eco*RI-*Xba*I fragment to generate plasmid pAtaA.

### Target mutagenesis of *ataA* by allelic marker exchange

To construct a Δ*ataA* mutant, we employed a double selection strategy using *sacB*, which is lethal to various Gram-negative bacteria in the presence of sucrose, as a negative selection marker. For construction of a suicide vector to knockout *ataA*, we amplified a 1,712 bp-internal fragment containing a part of *ataA* (corresponding to bases 9,060–10,771 of *ataA*) by PCR using primers ataA9060F/ataA10771R and cloned it into the pTA2 vector by using TArget clone -plus- (TOYOBO) according to the protocol provided by the manufacture. A selectable marker was inserted *in vitro* using the EZ:TN <TET-1> insertion kit (Epicentre Biotechnologies), and the <TET-1> insertion site was confirmed by sequencing. The *ataA* fragment integrated by <TET-1> was amplified by PCR using primers infusion-ataA9060F/infusion-ataA10771R and cloned into the *Bam*HI site of pJQ200sk by using the In-Fusion 2.0 Dry-down PCR Cloning kit (Clontech). The resultant suicide vector was transferred into Tol 5 cells from *E. coli* S17-1 via bacterial conjugation, and Gm^R^, Tc^R^, Suc^S^ (sucrose sensitive) colonies were selected on a BS agar plate containing 100 µg/ml gentamicin and 10 µg/ml tetracycline, and supplemented with toluene. Following, positive selection of allelic exchange mutants was performed on a BS agar plate containing 5% sucrose supplemented with toluene. Inactivation of *ataA* and the antibiotic resistance of the allelic exchange mutants were confirmed by PCR and the plating test (Gm^S^, Tc^R^, Suc^R^ (sucrose resistant)), respectively.

### Protein manipulation

Preparation of nanofiber proteins was performed as described previously [24]. For the preparation of outer membrane proteins (OMPs), bacterial cells were grown to the stationary phase, aerobically in 100 ml of LB medium for 6 h at 28°C (Tol 5 and its derivatives) or for 12 h at 30°C (ADP1 and its transformants), in the presence of 0.5% arabinose, with shaking at 115 rpm. The cultures (OD_660_ = 2.1) were harvested by centrifugation at 6,000 g for 10 min, washed with 50 mM phosphate buffer (pH 7.4), and then resuspended in 10 ml of the same buffer. The cells were lysed by sonication and centrifuged at 6,000 g for 10 min to remove unlysed cells and debris. The supernatant was centrifuged at 100,000 g for 1 h to pellet total membrane proteins which were then resuspended in a 0.5% *N*-lauroylsarcosine solution and incubated at room temperature for 30 min to lyse inner membrane proteins. The sample was centrifuged again at 100,000 g for 1 h and OMPs were collected as a pellet which was resuspended in 50 mM phosphate buffer (pH 7.4) or directly lysed in SDS-PAGE sample buffer.

### Production of GST-AtaA fusion protein and generation of an anti-AtaA antibody

Recombinant AtaA_699–1014_ was expressed in *E. coli* as a glutathione *S*-transferase (GST) fusion protein to generate an anti-AtaA antibody. The DNA fragment encoding AtaA_699–1014_ was amplified by PCR with the primer set AtaA-stalkF/AtaA-stalkR. The PCR product was digested with *Bam*HI and *Not*I, ligated into the *Bam*HI-*Not*I site of the pGEX4T-2 plasmid (GE Healthcare), and *E. coli* BL21 was transformed with the constructed plasmid. The transformant was cultured at 37°C until an OD_600_ of 1.5 was reached, and the expression of the GST fusion gene was then induced by adding 0.1 mM IPTG, followed by incubation at 25°C for 3 h. The cells were harvested by centrifugation, resuspended in PBS containing 1 mM dithiothreitol, lysed by sonication, and centrifuged at 6,000 g for 10 min to remove the unlysed cells and debris. The GST fusion protein was purified from the supernatant using GSTrap FF (GE Healthcare). The purified protein was inoculated into rabbits for generating a polyclonal anti-AtaA antiserum and antibody.

### Western-blot and immunodetection

OMPs were separated on 7.5% acrylamide gels and transferred to PVDF membrane with a constant voltage of 50 V overnight at 10°C using a Criterion Trans-Blot Cell (Bio-Rad) and blotting buffer (25 mM Tris; 192 mM Glycine ; 0.1% SDS; 5% methanol). The blotted membrane was blocked using a 5% skim milk solution for 1 h at room temperature and then treated with anti-AtaA_699–1014_ antiserum at a 1∶50,000 dilution in 5% skim milk solution. The proteins bound by the antiserum were detected with a horseradish peroxidase-conjugated anti-rabbit antibody (GE Healthcare) at a 1∶10,000 dilution in 5% skim milk solution and visualized using ECL Western Blotting Detection Reagents (GE Healthcare).

### Flow cytometry

*Acinetobacter* strains were inoculated into 2 ml of LB medium and grown overnight. The overnight cell cultures were diluted to 1∶100 with 20 ml of fresh LB medium and grown aerobically to the stationary phase (around 2.1 of OD_660_) for 6 h at 28°C (Tol 5 and its derivatives), or for 12 h at 30°C (ADP1 and its transformants) in the presence of 0.5% arabinose, with shaking at 115 rpm. Cells were washed with PBS, fixed with 4% paraformaldehyde, washed twice with PBS, and resuspended in PBS. Samples were treated with anti-AtaA_699–1014_ antiserum at a 1∶10,000 dilution in PBS containing 0.05% Tween 20 (Calbiochem) for 30 min at room temperature. After incubation, cells were washed twice with NET buffer (150 mM NaCl, 5 mM EDTA, 50 mM Tris-HCl, 5% Triton X-100 (Calbiochem); pH 7.6), treated with Alexa Fluor 488 conjugated to anti-rabbit antibody (Cell Signaling Technology) at a 1∶500 dilution in NET buffer for 30 min at room temperature, and washed three times with NET buffer. Finally, the cells were resuspended in pure water, and surface-displayed AtaA was measured by flow cytometry on a FACSCanto II (Becton Dickinson). Histograms were created using WinMDI 2.9 (J. Trotter) software.

### Adherence and autoagglutination assays

Cells of *Acinetobacter* strains were grown by the same procedure as flow cytometry. *Y. enterocolitica* strains were cultured and treated as described above for the induction of *yadA*. The grown cells were suspended in BS-N medium [49] containing no carbon nor nitrogen sources and were subjected to the adherence and autoagglutination assays under the resting cell condition, as described previously [23]. The cell suspension was incubated at 28°C for 2 h in the adherence assay. In the autoagglutination assay, test tubes containing the cell suspension were left to stand at room temperature for 3 h (tube-settling assay) [15], [50].

### Electron microscopy

Nanofibers on cell surfaces were observed using transmission electron microscopy (TEM) after staining cells with 1% methylamine tungstate (MAT) as described previously [24]. For immunoelectron microscopy, anti-AtaA_699–1014_ antibody and colloidal gold conjugated with a goat anti-rabbit IgG were used for the immunolabeling of the AtaA fibers. Carbon-coated copper grids were hydrophilized using an ion bombarder (PIB-10, Vacuum Device). Specimen preparation was performed in a moisture box to avoid desiccation. For each sample, 1 ml of cell culture with an OD_660_ of 0.6 was placed into a 1.5 ml tube and centrifuged at 6,000 g for 1 min. The pellet was resuspended in phosphate buffered saline (PBS) after rinsing the cells twice with PBS. One drop of cell suspension was placed on the hydrophilized carbon-coated copper grid and allowed to stand for 5 min. The excess liquid was removed using filter paper and the grid was rinsed twice with PBS. For blocking, one drop of PBS containing 1% BSA was placed on the grid and left for 1 h. For detection of the nanofiber formed by AtaA, the grid was incubated with an anti-AtaA antibody at a 1∶100 dilution in PBS containing 1% BSA for 2 h. The grid was carefully washed with PBS to reduce nonspecific background and was then incubated with colloidal gold (10 nm in diameter) conjugated with a goat anti-rabbit IgG (EY laboratories) at a 1∶100 dilution in PBS containing 1% BSA for 1 h. After washing, the grid was stained with 1% MAT and observed under a TEM (Hitachi H-7100) operated at 100 kV.

## Supporting Information

Figure S1 **Sequencing strategy for** ***ataA*** **containing several highly conserved, long repeat sequences.** To determine the DNA sequence of the gene disrupted in the transposon Tn5-inserted mutant T1, nine DNA fragments were obtained by inverse PCR, southern hybridization, and normal PCR. Initially, the Tn5 insertion site was identified from the genomic DNA of T1 by southern hybridization with the Tn5 *tetA* gene as a DNA probe, and the 5-kb flanking DNA region of the Tn5 insertion site was then amplified by inverse PCR. As a result of sequencing this fragment with the primer walking method, an incomplete structural gene (*ataA*), which showed partial homology with TAAs, was identified. The overlapping regions containing the *ataA* gene were cloned as seven DNA fragments (three *Pst* I and four *Hin*d III fragments) by repeating southern hybridization. As these DNA fragments contained several long repeat sequences which disturbed sequencing with the primer walking method, deletion fragments were prepared using exonuclease III to avoid this problem, and their sequences were then determined. Finally, a DNA fragment containing full-length *ataA* was amplified by PCR to reconfirm the sequence with the primer walking method. The sequencing primers used in the repeat-rich regions were individually designed so that their 3′ terminal nucleotide was inconsistent with that of other repeat sequences. The 20 black flags in the PCR fragment show the positions of the primers that were used to confirm the sequence and the direction of the sequencing from the respective primers.
(TIF)

Figure S2 **Full-length AtaA amino acid sequence.** Long repeat sequences can be classified into six groups colored light blue (243 aa), orange (58 aa), pink (115 aa), blue (86 aa), light green (79 aa), and green (130 aa) which minimize non-conserved amino acid residues. The non-conserved residues in each group are indicated by characters in bold font. Underlined characters indicate the amino acid residues that were expressed as a recombinant protein for generating anti-AtaA antibody.
(TIF)

Figure S3 **Prediction of AtaA deficiency in coiled coils.** The full-length amino acid sequence of AtaA was analyzed using COILS (http://www.ch.embnet.org/software/COILS_form.html) to determine the coiled coil probability score using a MTIDK matrix and a 28 residue window. AtaA was compared with several typical TAAs: YadA of *Y. enterocolitica*, BadA of *Bartonella henselae*, UspA1 of *Moraxella catarrhalis*, and Hia of *Haemophilus influenza*. Although the coiled coil structure is abundant in most TAAs, this structure is infrequent in the N-terminal stalk region of AtaA.
(TIF)

Figure S4 **Confirmation of the specificity of an anti-AtaA antibody on immunoelectron microscopy.** *Acinetobacter* sp. Tol 5 T1 (T1) and Δ*ataA* mutant (Δ*ataA*) were observed by immunoelectron microscopy using anti-AtaA_699–1014_ antibody. No nanofibers including AtaA were observed on T1 cells grown on toluene. The antibodies did not bind to nanofibers, which were different from AtaA, on Δ*ataA* cells.
(TIF)

Figure S5 **Confirmation of the production of type 1 and Fil fimbrial component proteins by SDS-PAGE and their deduced fibers by TEM in the Δ** ***ataA*** **strain.** (**A**) Samples were prepared from WT and Δ*ataA* cells grown on toluene. Monomeric FimA protein was detected by SDS-PAGE, and the deduced type 1 fimbriae were observed on the cell surface of Δ*ataA*. (**B**) Samples were prepared from WT and Δ*ataA* cells grown on triacylglycerol. Monomeric FilA protein was detected by SDS-PAGE, and the deduced Fil fimbriae were observed on the cell surface of Δ*ataA*. The bands of FimA and FilA were previously identified by Edman degradation.
(TIF)

Figure S6 ***E. coli-Acinetobacter*** **shuttle plasmid vector, pARP3, constructed for the study.** *araC*-P*_BAD_*, arabinose-inducible promoter and regulator; *Acinetobacter* ori, replication origin in genus *Acinetobacter*; Gm^R^, gentamicin-resistance marker; P3-Ap^R^, ampicillin-resistance marker under the control of the P3 promoter which has high transcriptional activity in *Acinetobacter*; OriT, origin of transfer; *traJ*, conjugal transfer transcriptional regulator; OriV, p15A replication origin of *E. coli*.
(TIFF)

Table S1 **Primers used in this study (excluding those for DNA sequencing).**
(DOC)
