# Supplementary figures and images for: AtaA, a New Member of the Trimeric Autotransporter Adhesins from Acinetobacter sp. Tol 5 Mediating High Adhesiveness to Various Abiotic Surfaces
Source: PLoS One. 2012 Nov 14;7(11):e48830. doi: 10.1371/journal.pone.0048830 (PMC3498257; doi:10.1371/journal.pone.0048830)

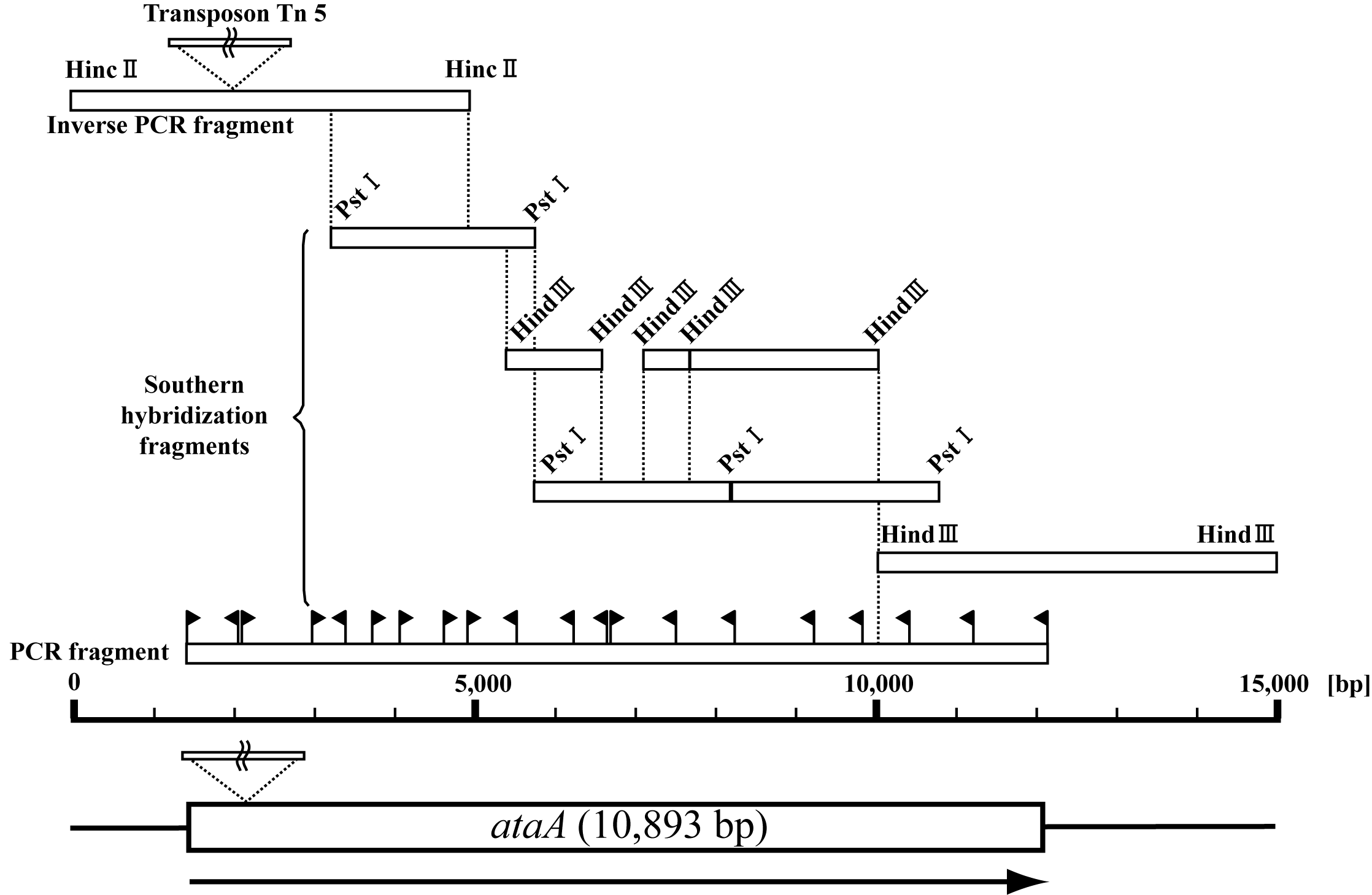

Supplement: Figure S1 — Sequencing strategy for ataA containing several highly conserved, long repeat sequences. To determine the DNA sequence of the gene disrupted in the transposon Tn5-inserted mutant T1, nine DNA fragments were obtained by inverse PCR, southern hybridization, and normal PCR. Initially, the Tn5 insertion site was identified from the genomic DNA of T1 by southern hybridization with the Tn5 tetA gene as a DNA probe, and the 5-kb flanking DNA region of the Tn5 insertion site was then amplified by inverse PCR. As a result of sequencing this fragment with the primer walking method, an incomplete structural gene (ataA), which showed partial homology with TAAs, was identified. The overlapping regions containing the ataA gene were cloned as seven DNA fragments (three Pst I and four Hind III fragments) by repeating southern hybridization. As these DNA fragments contained several long repeat sequences which disturbed sequencing with the primer walking method, deletion fragments were prepared using exonuclease III to avoid this problem, and their sequences were then determined. Finally, a DNA fragment containing full-length ataA was amplified by PCR to reconfirm the sequence with the primer walking method. The sequencing primers used in the repeat-rich regions were individually designed so that their 3′ terminal nucleotide was inconsistent with that of other repeat sequences. The 20 black flags in the PCR fragment show the positions of the primers that were used to confirm the sequence and the direction of the sequencing from the respective primers. (TIF) [file pone.0048830.s001.tif]

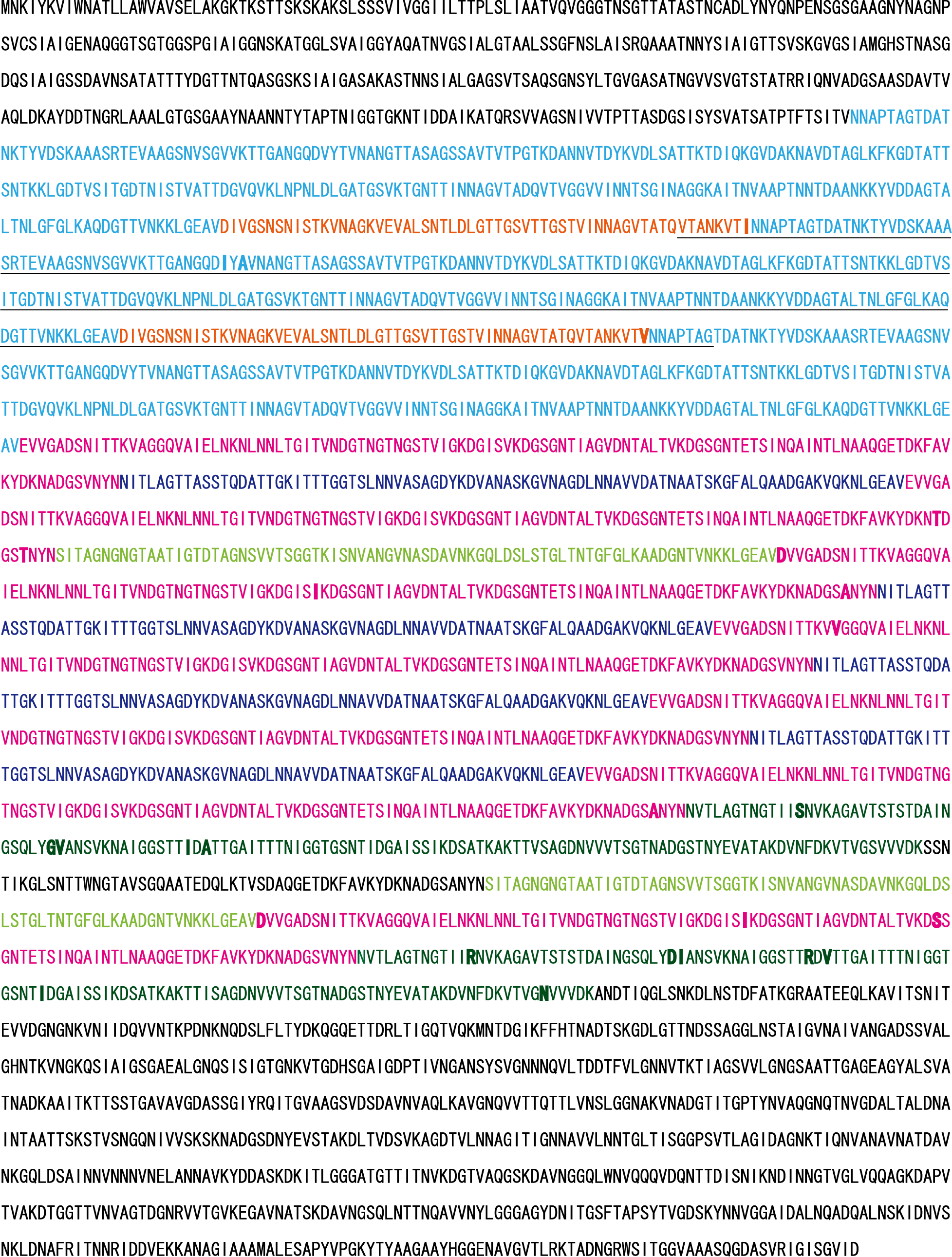

Supplement: Figure S2 — Full-length AtaA amino acid sequence. Long repeat sequences can be classified into six groups colored light blue (243 aa), orange (58 aa), pink (115 aa), blue (86 aa), light green (79 aa), and green (130 aa) which minimize non-conserved amino acid residues. The non-conserved residues in each group are indicated by characters in bold font. Underlined characters indicate the amino acid residues that were expressed as a recombinant protein for generating anti-AtaA antibody. (TIF) [file pone.0048830.s002.tif]

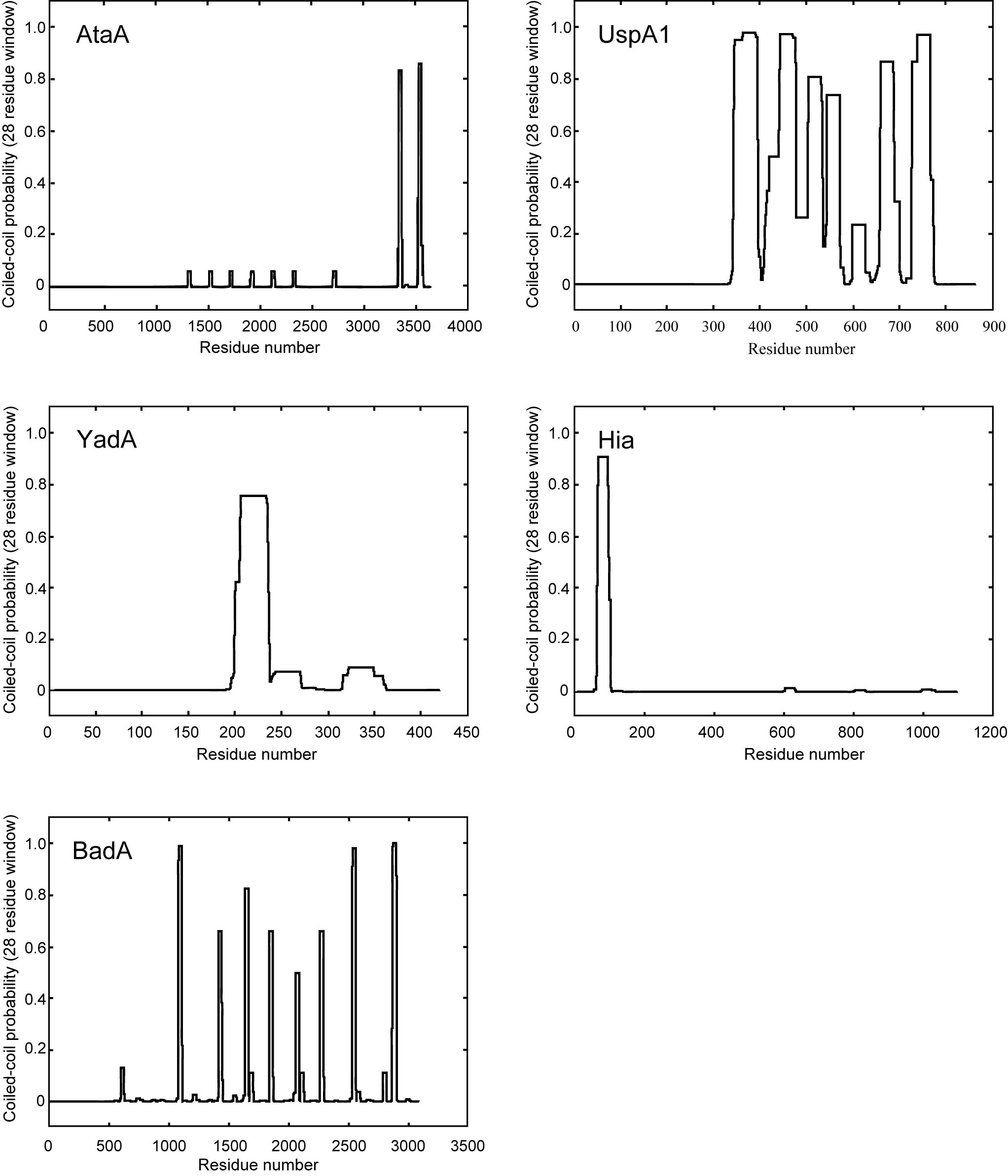

Supplement: Figure S3 — Prediction of AtaA deficiency in coiled coils. The full-length amino acid sequence of AtaA was analyzed using COILS (http://www.ch.embnet.org/software/COILS_form.html) to determine the coiled coil probability score using a MTIDK matrix and a 28 residue window. AtaA was compared with several typical TAAs: YadA of Y. enterocolitica, BadA of Bartonella henselae, UspA1 of Moraxella catarrhalis, and Hia of Haemophilus influenza. Although the coiled coil structure is abundant in most TAAs, this structure is infrequent in the N-terminal stalk region of AtaA. (TIF) [file pone.0048830.s003.tif]

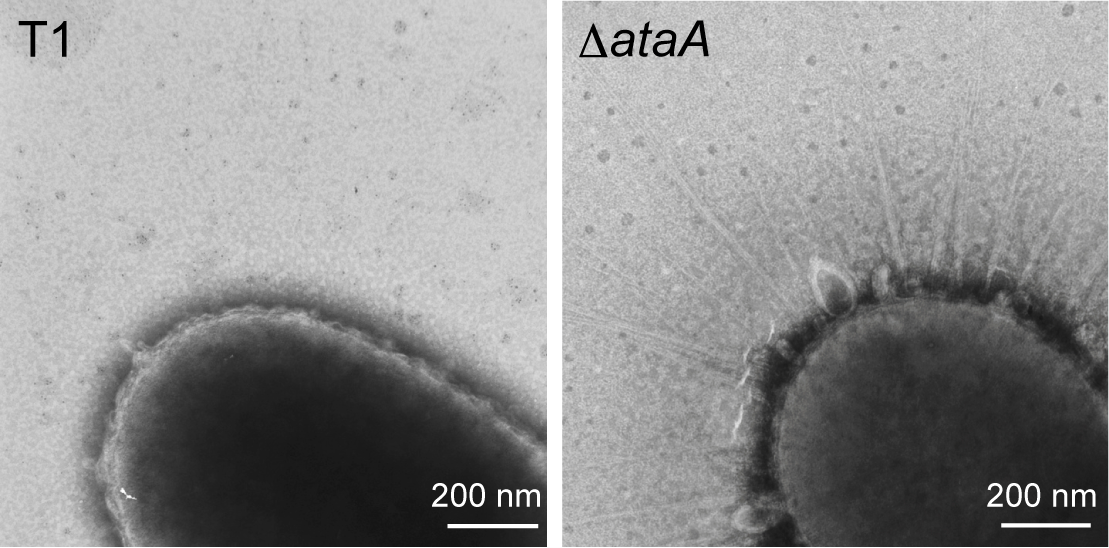

Supplement: Figure S4 — Confirmation of the specificity of an anti-AtaA antibody on immunoelectron microscopy. Acinetobacter sp. Tol 5 T1 (T1) and ΔataA mutant (ΔataA) were observed by immunoelectron microscopy using anti-AtaA699–1014 antibody. No nanofibers including AtaA were observed on T1 cells grown on toluene. The antibodies did not bind to nanofibers, which were different from AtaA, on ΔataA cells. (TIF) [file pone.0048830.s004.tif]

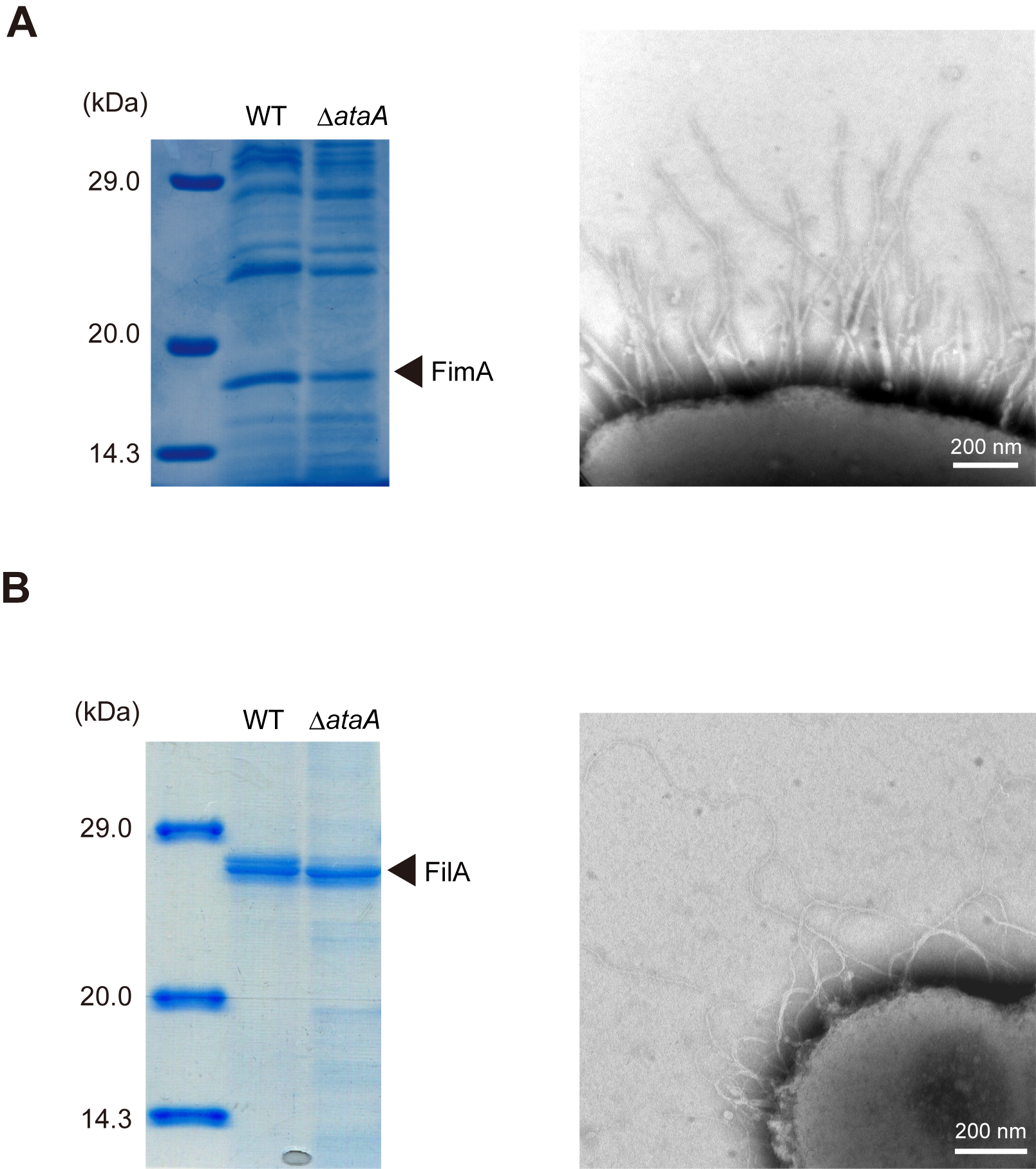

Supplement: Figure S5 — Confirmation of the production of type 1 and Fil fimbrial component proteins by SDS-PAGE and their deduced fibers by TEM in the Δ ataA strain. (A) Samples were prepared from WT and ΔataA cells grown on toluene. Monomeric FimA protein was detected by SDS-PAGE, and the deduced type 1 fimbriae were observed on the cell surface of ΔataA. (B) Samples were prepared from WT and ΔataA cells grown on triacylglycerol. Monomeric FilA protein was detected by SDS-PAGE, and the deduced Fil fimbriae were observed on the cell surface of ΔataA. The bands of FimA and FilA were previously identified by Edman degradation. (TIF) [file pone.0048830.s005.tif]

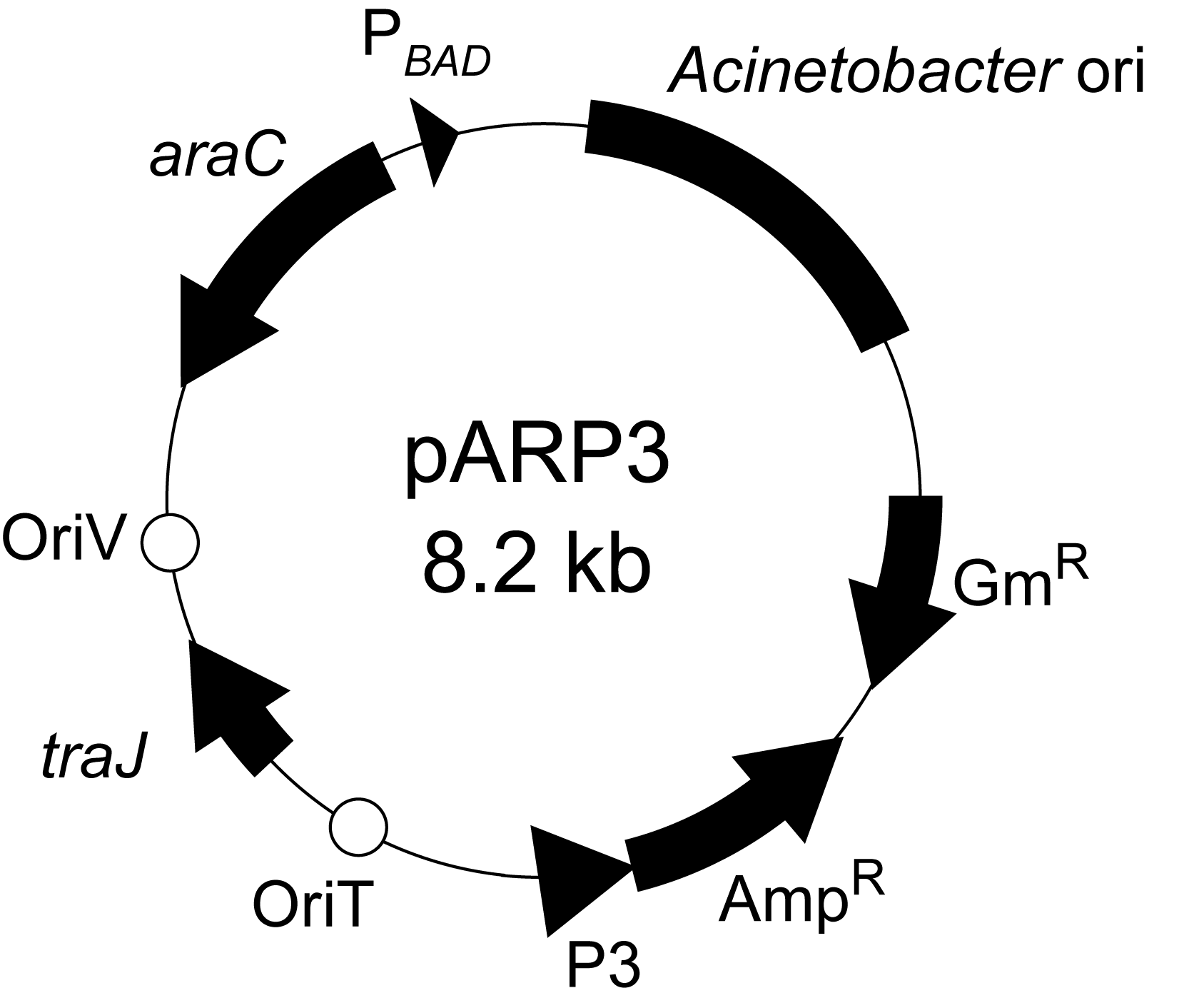

Supplement: Figure S6 — E. coli-Acinetobacter shuttle plasmid vector, pARP3, constructed for the study. araC-PBAD, arabinose-inducible promoter and regulator; Acinetobacter ori, replication origin in genus Acinetobacter; GmR, gentamicin-resistance marker; P3-ApR, ampicillin-resistance marker under the control of the P3 promoter which has high transcriptional activity in Acinetobacter; OriT, origin of transfer; traJ, conjugal transfer transcriptional regulator; OriV, p15A replication origin of E. coli. (TIFF) [file pone.0048830.s006.tiff]
