# Supplementary material for: AtaA, a New Member of the Trimeric Autotransporter Adhesins from Acinetobacter sp. Tol 5 Mediating High Adhesiveness to Various Abiotic Surfaces
Source: PLoS One. 2012 Nov 14;7(11):e48830. doi: 10.1371/journal.pone.0048830 (PMC3498257; doi:10.1371/journal.pone.0048830)
Supplement: Table S1 — Primers used in this study (excluding those for DNA sequencing). (DOC) [file pone.0048830.s007.doc]

| **Table S1. Primers used in this study (excluding those for DNA sequencing)** | |
| --- | --- |
| Primer | Sequence (5'→3')^†^ |
| AtaA-stalkF | CGCGGATCCGTTACCGCAAACAAAGTCACAATA |
| AtaA-stalkR | ATTGCGGCCGCACCTGCTGTTGGTGCATTATTAAC |
| *Acineto*-oriF | ACATGCATGCGATCGTAGAAATATCTATG |
| *Acineto*-oriR | TGTAGCATGCGGATTTTAACATTTTGCGTTGTTC |
| dele-sacBF | CGACAGCTGTAAAAACGCAAAAGAAAATGCCGA |
| dele-sacBR | CGACAGCTGAATACTGTTGCTTGTTTTGCAAAC |
| P3-ApF | CGACAGCTGGATCAATCTTTAAAATTTTATCTAAAG |
| P3-ApR | CGACAGCTGCAAACAAACCACCGCTGGTAG |
| attB1-SD-AtaAF | AAAAAGCAGGCTGAATTCATCTCCTAAGGAAAAGCGATATG |
| attB2-STOP-AtaAR | AGAAAGCTGGGTCTAGAGAATTAGTCAATCACACCGCTG |
| attB1-adapter | GGGGACAAGTTTGTACAAAAAAGCAGGCT |
| attB2-adapter | GGGGACCACTTTGTACAAGAAAGCTGGGT |
| ataA9060F | CAATTGCAATTGGTTCTGGTGC |
| ataA10771R | ACGTAAAGTCACACCTACCGC |
| Infusion-ataA9060F | TAGAACTAGTGGATCCCAATTGCAATTGGTTCTGGTGC |
| Infusion-ataA10771R | GCAGCCCGGGGGATCCACGTAAAGTCACACCTACCGC |

^†^Underlined nucleotides represent *Bam*HІ (GGATCC), *Not*І (GCGGCCGC), *Sph*І (GCATGC), *Pvu*ІІ (CAGCTG), *Eco*RІ (GAATTC), and *Xba*І (TCTAGA) restriction sites.
